# A Two-Filter Approach for State Estimation Utilizing Quantized Output Data

**DOI:** 10.3390/s21227675

**Published:** 2021-11-18

**Authors:** Angel L. Cedeño, Ricardo Albornoz, Rodrigo Carvajal, Boris I. Godoy, Juan C. Agüero

**Affiliations:** 1 Departamento Electrónica, Universidad Técnica Federico Santa María (UTFSM), Av. España 1680, Valparaíso 2390123, Chile; ricardo.albornoz.13@sansano.usm.cl (R.A.); juan.aguero@usm.cl (J.C.A.); 2 Advanced Center for Electrical and Electronic Engineering, AC3E, Gral. Bari 699, Valparaíso 2390136, Chile; 3 Escuela de Ingeniería Eléctrica, Pontificia Universidad Católica de Valparaíso, Av. Brasil 2147, Valparaíso 2374631, Chile; rodrigo.carvajal@pucv.cl; 4 Department of Mechanical Engineering, Boston University, Boston, MA 02215, USA; bgodoy@bu.edu

**Keywords:** state estimation, quantized data, Gaussian sum filtering, Gaussian sum smoothing, Gauss–Legendre quadrature

## Abstract

Filtering and smoothing algorithms are key tools to develop decision-making strategies and parameter identification techniques in different areas of research, such as economics, financial data analysis, communications, and control systems. These algorithms are used to obtain an estimation of the system state based on the sequentially available noisy measurements of the system output. In a real-world system, the noisy measurements can suffer a significant loss of information due to (among others): (i) a reduced resolution of cost-effective sensors typically used in practice or (ii) a digitalization process for storing or transmitting the measurements through a communication channel using a minimum amount of resources. Thus, obtaining suitable state estimates in this context is essential. In this paper, Gaussian sum filtering and smoothing algorithms are developed in order to deal with noisy measurements that are also subject to quantization. In this approach, the probability mass function of the quantized output given the state is characterized by an integral equation. This integral was approximated by using a Gauss–Legendre quadrature; hence, a model with a Gaussian mixture structure was obtained. This model was used to develop filtering and smoothing algorithms. The benefits of this proposal, in terms of accuracy of the estimation and computational cost, are illustrated via numerical simulations.

## 1. Introduction

It is well known that discrete-time dynamical systems can be described as first-order difference equations relating internal variables called states [1]. State estimation is a scientific discipline that studies methodologies and algorithms for estimating the state of dynamical systems from input–output measurements [2,3]. There are a variety of applications that use state estimation, such as control [4,5,6,7], parameter identification [8,9,10], power systems [11,12], fault detection [13,14,15,16,17], prognosis [18,19], cyber–physical systems [20], hydrologic and geophysical data assimilation [21,22], maritime tracking [23], consensus-based state estimation using wireless sensor networks [24,25,26], navigation systems [27], and transportation and highway traffic management [28,29,30], to mention a few. Depending on the measurements that are used, two algorithms of state estimation can be distinguished: filtering and smoothing. Filtering algorithms estimate the current state using measurements up to the current instant, and smoothing algorithms estimate the state at some time in the past using measurement up to the current instant [23,31].

In general, the experimental noisy data can suffer a significant loss of information introduced by low-resolution and cost-effective sensors [32] in the digitalization process [33]. Typically, the digitalization process encompasses a process known as *quantization*. Quantization is a nonlinear map that partitions the whole signal space and represents all of the values in each subspace by a single one [32]. In spite of the loss of information, the benefits of quantization have led to a number of applications in which quantized measurements arise. This occurs due to fundamental limitations on measuring equipment and bandwidth resources [34], digital and analog converters [35], and experimental designs where it is necessary to quantize the data in order to store it or minimize communication resource utilization [36]. In particular, estimation problems utilizing quantized measurements arise in networked control over limited-/finite-capacity communication channels, where usually, encoder–decoder state estimation schemes are used [37,38,39,40]. In addition, in order to deal with uncertain dynamic systems, robust estimation algorithms have also been developed; see, e.g., [37,38,41].

Currently, state estimation from quantized data has gained significant attention in a growing number of applications such as fault detection [42,43], networked control [42,44,45], and system identification [35,46,47,48]. For instance, in [49,50], Kalman-based state estimation algorithms were developed using multiple sensors for distributed systems. In [24], a Kalman smoothing algorithm was developed for any-time minimum-mean-squared error optimal-consensus-based state estimation using wireless sensor networks. In [27], an online smoothing algorithm was developed to estimate the positional and orientation parameters of integrated navigation systems utilizing a low-cost microelectromechanical system inertial sensor in near-real time.

**Figure 1:**
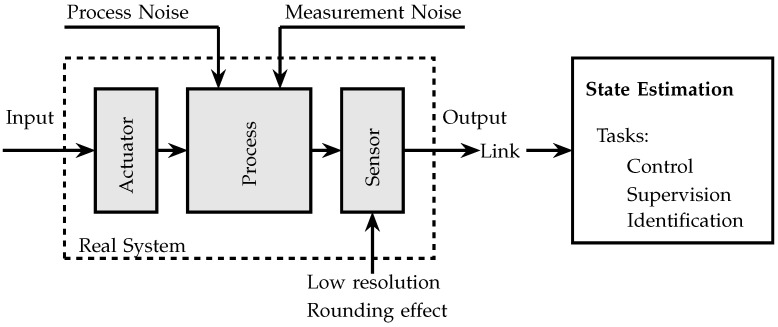
Diagram of a real dynamic system with quantized data.

In Figure 1, a usual representation of a process is shown, which is defined by the interconnection of three blocks: (1) an actuator, (2) a process, and (3) a sensor. This representation includes a link that can be a communication channel and a base station. The actuator input usually comes from a control system, and the output of the process is measured with noise by a sensor. The sensor introduces quantization to the noisy measurements. This representation has been used for state estimation and control in a microgrid with multiple distributed energy resources, where a dequantizer is used to reconstruct the received signal and then perform the standard Kalman filter [51]. In [42], a fault isolation filter for a discrete-time networked control system with multiple faults was developed using the Kalman filter, where the sensor measurements were transmitted only when the output signal was greater than a threshold. The authors in [52] dealt with a similar structure to the one in Figure 1 to estimate the vehicle sideslip angle of an in-vehicle networked system with sensor failure, dynamic quantization, and data dropouts. For more examples, see, e.g., [15,53].

For linear systems and Gaussian noises (without quantization), the optimal estimator of the system state is the celebrated Kalman filter [1] and smoother [22]. However, in the most general case, i.e., nonlinear systems and non-Gaussian noises, it is not possible to obtain an optimal estimator because the computation of some integrals in the filtering and smoothing equations is difficult or the integrals are just intractable. As mentioned above, the quantization process is a nonlinear map that results in a significant loss of information on the system dynamics, which produces a biased state estimation and incorrect characterization of the filtering and smoothing probability density functions (PDFs). In this context, several suboptimal filtering and smoothing algorithms for state-space systems with quantized data have been developed, for instance, standard- [49,54], unscented- [55], and extended- [36] Kalman filters for quantized data, in which some structural elements of the state-space models and the quantizer are exploited. Sequential Monte Carlo methods have been also used for filtering and smoothing with quantized data, where complex integrals are approximated by a set of weighted samples called particles [31], which define (approximately) a desired PDF. However, the most common difficulty in these methods is dealing with the quantizer model. Some approaches have been proposed for this purpose such as gradient-based approximation of the quantizer [56] or modeling the quantizer as uniformly distributed additive noise [32,57]. In [58], an approximation of the integral in the non-Gaussian probability mass function (PMF) of the quantized output given the state was proposed by using Gaussian quadrature rules in order to deal with binary data. This approximation naturally yields an explicit Gaussian mixture model (GMM) form for the PMF of the quantized output. In this paper, the approximation of this integral was extended by considering a more general (finite- and infinite-level) quantizer, and it was used to develop Gaussian sum filtering and smoothing algorithms.

### Main Contributions

The main contributions of this work are:

1. Developing an explicit model (of the GMM form) for the PMF of the quantized output considering a finite- and infinite-level quantizer, to solve in closed-form filtering and smoothing recursions in a Bayesian framework;
2. Designing Gaussian sum filtering and smoothing algorithms to deal with quantized data, providing closed expressions for the state estimates and for the filtering and smoothing PDFs.

The filtering algorithm for quantized data presented in this paper includes, as a particular case, the filtering algorithm presented in [58], where only the case of binary data was considered. Additionally, the smoothing algorithm presented here, based on the approximation of the PMF of the quantized output given the state, is completely novel. The remainder of the paper is as follows: In Section 2, the problem of interest is defined. In Section 3, the characterization and approximation of the PMF of the quantized data given the state are presented, and using this explicit model, the Gaussian sum filtering and smoothing algorithms are developed. In Section 4, some examples are presented to show the benefits of this proposal in terms of accuracy and computational cost. Finally, in Section 5, conclusions are presented.

## 2. Statement of the Problem

### 2.1. System Model

**Figure 2:**
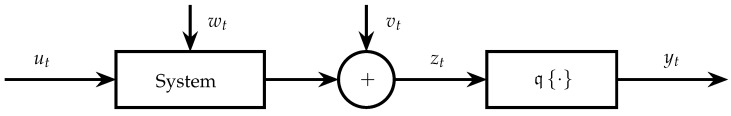
State-space model with quantized output.

Consider the state-space model for a discrete-time linear-time-invariant system with quantized output (see Figure 2):

(1)xt+1=Axt+But+wt,(2)zt=Cxt+Dut+vt,(3)yt=qzt,

where $x_{t}\in R^{n}$ is the state vector, $z_{t}\in R$ is the nonquantized output, $y_{t}\in R$ is the quantized output, and $u_{t}\in R^{m}$ is the input of the system. The matrices are $A\in R^{n\times n}$, $B\in R^{n\times m}$, $C\in R^{1\times n}$, and $D\in R^{1\times m}$. The nonlinear map $q\left\{\cdot \right\}$ is the quantizer. The state noise $w_{t}\in R^{n}$ and the output noise $v_{t}\in R$ are zero-mean white Gaussian noises with covariance matrix *Q* and *R*, respectively. The system in (1)–(2) can be described using the state transition and conditional nonquantized output probability distributions:

(4)p(x1)=Nx1(μ1,P1),(5)p(xt+1|xt)=Nxt+1(Axt+But,Q),(6)p(zt|xt)=Nzt(Cxt+Dut,R),

where $N_{x}\left(\mu ,P\right)$ represents a PDF corresponding to a Gaussian distribution with mean μ and the covariance matrix *P* of the variable *x*. The initial condition $x_{1}$, the model noise $w_{t}$, and the measurement noise $v_{t}$ are statistically independent random variables.

### 2.2. Quantizer Model

Let the quantizer $q\left\{\cdot \right\}:R\to V$ be a map from the real line defined by the set of intervals $\left\{J_{i}\subset R:i\in I\right\}$ to the finite or countable infinite set $V=\left\{\beta_{i}\in R:i\in I\right\}$ [59], where I is a set of indices defined by the quantizer type. In this work, two quantizers were considered. The first an infinite-level quantizer, in which the output of the quantizer has infinite (countable) levels of quantization corresponding to the indices’ set:

(7) $$\begin{array}{c} I=\left\{\ldots ,1,2,\ldots ,L,\ldots \right\}. \end{array}$$

**Figure 3:**
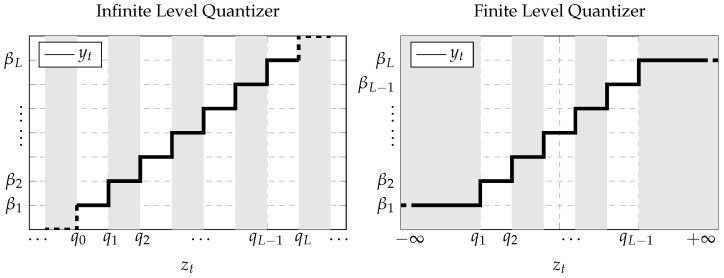
Representation of the (**left**) uniform infinite- and (**right**) finite-level quantizers defined in terms of the quantized values $\beta_{i}$ and the intervals $J_{i}$.

The definition of the infinite-level quantizer is as follows (see Figure 3 (left)):

(8) $$q\left\{z_{t}\right\}=\begin{array}{ccccc} \beta_{i} & \; & \mathrm{if} & \; & z_{t}\in J_{i}, \end{array}\;i\in I,$$

where the sets $J_{i}=\left\{z_{t}:q_{i-1}\leq z_{t}<q_{i}\right\}$ are disjoint intervals and each $\beta_{i}$ is the value that the quantizer takes in the region $J_{i}$. The second is a finite-level quantizer, in which the output of the quantizer is limited to a minimum and maximum values (saturated quantizer) corresponding to the indices’ set:

(9) $$\begin{array}{c} I=\left\{1,2,\ldots ,L-1,L\right\}. \end{array}$$

The definition of the finite-level quantizer (see Figure 3 (right)) is similar to (8), where I is defined in (9), $J_{1}=\left\{z_{t}:z_{t}<q_{1}\right\}$, $J_{L}=\left\{z_{t}:q_{L-1}\leq z_{t}\right\}$, and $J_{i}=\left\{z_{t}:q_{i-1}\leq z_{t}<q_{i}\right\}$ with i=2…,L−1.

### 2.3. Problem Definition

The problem of interest can be defined as follows: Given the available data $u_{1:N}=\left\{u_{1},u_{2},\ldots ,u_{N}\right\}$ and $y_{1:N}=\left\{y_{1},y_{2},\ldots ,y_{N}\right\}$, where *N* is the data length, obtain the filtering and smoothing PDFs of the state given the quantized measurements, $p(x_{t}|y_{1:t})$ and $p(x_{t}|y_{1:N})$, respectively the state estimators:

(10)x^t|t=Ext|y1:t,(11)x^t|N=Ext|y1:N,

and the corresponding covariance matrices of the estimation error:

(12)Σt|t=E(xt−x^t|t)(xt−x^t|t)T|y1:t,(13)Σt|N=E(xt−x^t|N)(xt−x^t|N)T|y1:N,

where t≤N and $E\left\{x|y\right\}$ denotes the conditional expectation of *x* given *y*.

## 3. Gaussian Sum Filtering and Smoothing for Quantized Data

Here, the Gaussian sum filtering and smoothing algorithms for quantized output data are explained in detail.

### 3.1. Gaussian Mixture Models

Gaussian mixture models refer to a convex combination of Gaussian densities corresponding to a random variable $\zeta \in R^{n}$. Then, the PDF can be written as [60]: $p\left(\zeta \right)=\sum_{i=1}^{K}\varphi_{i}N_{\zeta }\left(\upsilon_{i},\Gamma_{i}\right)$ subject to $\varphi_{i}>0$ and $\sum_{i=1}^{K}\varphi_{i}=1$, where $\varphi_{i}$ is the *i*th mixing weight, $\upsilon_{i}\in R^{n}$ is the *i*th mean, and $\Gamma_{i}\in R^{n\times n}$ is the *i*th covariance matrix. GMMs are used in a variety of applications to approximate non-Gaussian densities [61,62,63] and filtering and smoothing [64,65], to mention a few.

### 3.2. General Bayesian Framework

The well-known equations for Bayesian filtering (see, e.g., [31]) are given by:

(14)p(xt|y1:t)=p(yt|xt)p(xt|y1:t−1)p(yt|y1:t−1),(15)p(xt+1|y1:t)=∫p(xt+1|xt)p(xt|y1:t)dxt,

where $p(x_{t}|y_{1:t})$ and $p(x_{t+1}|y_{1:t})$ are the measurement and time-update equations, respectively. The PDF $p(x_{t+1}|x_{t})$ is obtained from the model in (5), and $p(y_{t}|y_{1:t-1})$ is a normalization constant. On the other hand, the well-known formula for Bayesian smoothing (see, e.g., [31]) is defined by:

(16) $$p\left(x_{t}|y_{1:N}\right)=p\left(x_{t}|y_{1:t}\right)\;\int \frac{p\left(x_{t+1}|y_{1:N}\right)p\left(x_{t+1}|x_{t}\right)}{p(x_{t+1}|y_{1:t})}dx_{t+1}.$$

However, the PDF in (16) is difficult to compute because of the division by a non-Gaussian density. This difficulty was overcome in [64] where the smoothing equations were separated in a two-fold formula filter: (i) the first formula, called the *backward filter*, defined by the following recursion:

(17)p(yt+1:N|xt)=∫p(yt+1:N|xt+1)p(xt+1|xt)dxt+1,(18)p(yt:N|xt)=p(yt|xt)p(yt+1:N|xt),

where $p(y_{t+1:N}|x_{t})$ and $p(y_{t:N}|x_{t})$ are the backward prediction and the backward- measurement-update equations, respectively, and (ii) the second formula, given by:

(19) $$p\left(x_{t}|y_{1:N}\right)=\frac{p\left(x_{t}|y_{1:t-1}\right)p\left(y_{t:N}|x_{t}\right)}{p(y_{t:N}|y_{1:t-1})},$$

where $p(x_{t}|y_{1:t-1})$ is the time-update equation from the filtering step, $p(y_{t:N}|y_{1:t-1})$ is a normalization constant, and $p(y_{t:N}|x_{t})$ is obtained using the backward recursion given in (17) and (18).

Due to the non-Gaussianity of $p(y_{t}|x_{t})$, the integrals in both the filtering and backward-filtering algorithms in (15) and (17), respectively, are difficult to compute or intractable. Widely used methods to deal with these integrals are Monte-Carlo-based algorithms, such as particle filtering and smoothing; see, e.g., [31,66]. These methods represent the posterior distributions $p(x_{t}|y_{1:t})$ and $p(x_{t}|y_{1:N})$ by a set of weighted random samples. However, in general, they exhibit a growing computational complexity as the model dimension increases. Here, an alternative method to compute the PMF $p(y_{t}|x_{t})$ using Gauss–Legendre quadrature is proposed. This procedure results in a GMM form. Hence, the integrals in both the filtering and backward filtering in (15) and (17), respectively, can be computed in closed form.

**Remark** **1.** *Notice that since $y_{t}$ is a discrete random variable, the measurement-update equation in* (14) *and the backward-measurement-update equation in* (18) *comprise PDFs and a PMF. Hence, generalized probability density functions are used here; see, e.g., [67].*

### 3.3. Computing an Explicit Model of $p(y_{t}|x_{t})$

**Figure 4:**
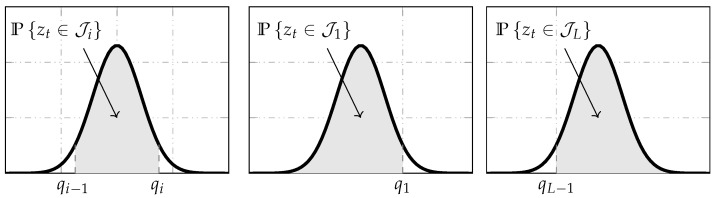
The shaded area represents the probability of $y_{t}$ taking a value $\beta_{i}$ that is equal to the probability of $z_{t}$ belonging to set $\in J_{i}$. Here, $P\left\{\cdot \right\}$ denotes probability.

From (8), it is observed that the output $z_{t}\in J_{i}$ is mapped to a single value $\beta_{i}$. Then, the probability that $y_{t}$ takes the value $\beta_{i}$ is the same as the probability of $z_{t}$ belonging to set $J_{i}$, as shown in Figure 4. Notice that the quantizer regions $J_{i}$ include finite and semi-infinite intervals.

In the following theorem, the characterization of $p(y_{t}|x_{t})$ is formalized via the computation of the probability $P\left\{z_{t}\in J_{i}\right\}$ shown in Figure 4 through the integral definition of probabilities [68]. Therefore, the approximation of this integral by using the Gauss–Legendre quadrature rule is presented (see, e.g., [69]).

**Theorem** **1.** *Consider the system* (1)–(3), *the infinite- and finite-level quantizers defined in* (8). *Then, the PMF of the discrete random variable $y_{t}$ given the state $x_{t}$ is given by:*

(20) $$p\left(y_{t}|x_{t}\right)=\int_{a_{t}}^{b_{t}}N_{v_{t}}\left(0,R\right)dv_{t},$$

*where $a_{t}$ and $b_{t}$ are functions of the boundary values of each region of the quantizers and are defined in Table 1. In addition, the integral in* (20) *can be approximated using the Gauss–Legendre quadrature rule, yielding:*

(21) $$p\left(y_{t}|x_{t}\right)\approx \sum_{\tau =1}^{K}\varsigma_{t}^{\tau }N_{\eta_{t}^{\tau }}\left(Cx_{t}+Du_{t}+\mu_{t}^{\tau },R\right),$$

*where K is the number of points from the Gauss–Legendre quadrature rule (defined by the user), $\varsigma_{t}^{\tau }$, $\eta_{t}^{\tau }$, and $\mu_{t}^{\tau }$ are defined in Table 1, and $\omega_{\tau }$ and $\psi_{\tau }$ are weights and points defined by the quadrature rule, given in, e.g., [69].*

**Proof.**  From the infinite- and finite-level quantizers, it is observed that the random variable $y_{t}$ can only take the values $\beta_{i}$ with *i* in the indices’ sets given in (7) and (9). Then, the probability of $y_{t}=\ldots ,\beta_{1},\beta_{2},\ldots ,\beta_{L},\ldots$ or $y_{t}=\beta_{1},\beta_{2},\ldots ,\beta_{L-1},\beta_{L}$ is the same as the probability that the random variable $z_{t}$ belongs to the sets $J_{i}$. This probability can be obtained from the distribution function as follows:

(22) $$P\left\{y_{t}=\beta_{i}|x_{t}\right\}=P\left\{z_{t}\in J_{i}|x_{t}\right\},$$

where $P\left\{\cdot \right\}$ denotes probability. Considering the infinite-level quantizer and the output equation in (2), the following expressions are obtained for $y_{t}=\beta_{i}$ with i=…,1,2…,L,…:

(23) $$\begin{array}{cc} P\left\{y_{t}=\beta_{i}|x_{t}\right\} & =P\left\{q_{i-1}\leq z_{t}<q_{i}|x_{t}\right\},\\ \; & =P\left\{a_{t}\leq v_{t}<b_{t}|x_{t}\right\}, \end{array}$$

where $a_{t}=q_{i-1}-Cx_{t}-Du_{t}$ and $b_{t}=q_{i}-Cx_{t}-Du_{t}$. Additionally, for the finite-level quantizer, (23) holds for $y_{t}=\beta_{i}$ with i=2,…,L−1, and for $y_{t}=\beta_{1}$ and $y_{t}=\beta_{L}$, the following holds:

(24)Pvt<bt|xtifyt=β1,(25)Pvt≥at|xtifyt=βL,

where $b_{t}=q_{1}-Cx_{t}-Du_{t}$ and $a_{t}=q_{L-1}-Cx_{t}-Du_{t}$. Then, using the fact that $p\left(v_{t}\right)=N_{v_{t}}\left(0,R\right)$ and using (23)–(25), the integral in (20) can be obtained, where the integral limits are given in Table 1.

On the other hand, the Gauss–Legendre quadrature for approximating a definite integral over the interval $\left[-1,1\right]$ is given by:

(26) $$\int_{-1}^{1}f\left(r\right)dr\approx \sum_{\tau =1}^{K}\omega_{\tau }f\left(\psi_{\tau }\right),$$

where $\omega_{\tau }$ and $\psi_{\tau }$ are the quadrature weights and the roots of the order *K* Legendre polynomial, respectively; see, e.g., [69]. Notice that the integral over $\left[a_{t},b_{t}\right]$ can be mapped onto the interval $\left[-1,1\right]$ using:

(27) $$\int_{a_{t}}^{b_{t}}f\left(r\right)dr=\frac{b_{t}-a_{t}}{2}\int_{-1}^{1}f\left(\frac{b_{t}-a_{t}}{2}r+\frac{b_{t}+a_{t}}{2}\right)dr,$$

and using the definition in (26), this integral is approximated by:

(28) $$\int_{a_{t}}^{b_{t}}f\left(r\right)dr\approx \frac{b_{t}-a_{t}}{2}\sum_{\tau =1}^{K}\omega_{\tau }f\left(\frac{b_{t}-a_{t}}{2}\psi_{\tau }+\frac{b_{t}+a_{t}}{2}\right).$$

Defining $a_{t}=q_{i-1}-Cx_{t}-Du_{t}$, $b_{t}=q_{i}-Cx_{t}-Du_{t}$, and $f\left(v_{t}\right)=N_{v_{t}}\left(0,R\right)$ with i=…,1,2…,L,…, the approximation of $p(y_{t}|x_{t})$ given in (21) for the infinite-level quantizer is derived.

For the finite-level quantizer, (28) holds for $y_{t}=\beta_{i}$ with i=2,…,L−1. Then, the approximation of $p(y_{t}|x_{t})$ for $y_{t}=\beta_{1}$ and $y_{t}=\beta_{L}$ is defined. First, it is observed that the integral over the semi-infinite interval $\left[a_{t},\infty \right)$ can be mapped onto the interval (0,1] using $r=a_{t}+\left(1-\xi \right)/\xi$, so that:

(29) $$\int_{a_{t}}^{\infty }f\left(r\right)dr=\int_{0}^{1}f\left(a_{t}+\frac{1-\xi }{\xi }\right)\frac{d\xi }{\xi^{2}}.$$

Then, using an appropriate change of variable, it can be mapped onto the interval (−1,1], yielding:

(30) $$\int_{a_{t}}^{\infty }f\left(r\right)dr=\int_{-1}^{1}f\left(a_{t}+\frac{1-s}{1+s}\right)\frac{2ds}{{\left(1+s\right)}^{2}}.$$

Using the Gauss–Legendre approximation given in (26), the approximation of $p(y_{t}|x_{t})$ for $y_{t}=\beta_{L}$ is obtained as follows:

(31) $$\int_{a_{t}}^{\infty }f\left(r\right)dr\approx \sum_{\tau =1}^{K}\omega_{\tau }f\left(a_{t}+\frac{1-\psi_{\tau }}{1+\psi_{\tau }}\right)\frac{2}{{\left(1+\psi_{\tau }\right)}^{2}}.$$

Defining $a_{t}=q_{L-1}-Cx_{t}-Du_{t}$, b=∞, and $f\left(v_{t}\right)=N_{v_{t}}\left(0,R\right)$, (21) is obtained. A similar procedure for the integral over the semi-infinite interval $\left(-\infty ,b_{t}\right]$ can be applied using $r=b_{t}-\left(1-\xi \right)/\xi$ to find the approximation of $p(y_{t}|x_{t})$ for $y_{t}=\beta_{1}$, as follows:

(32) $$\int_{-\infty }^{b_{t}}f\left(r\right)dr\approx \sum_{\tau =1}^{K}\omega_{\tau }f\left(b_{t}-\frac{1-\psi_{\tau }}{1+\psi_{\tau }}\right)\frac{2}{{\left(1+\psi_{\tau }\right)}^{2}}.$$

Defining $a_{t}=-\infty$, $b_{t}=q_{1}-Cx_{t}-Du_{t}$, and $f\left(v_{t}\right)=N_{v_{t}}\left(0,R\right)$, (21) is obtained. This completes the proof.   □

**Remark** **2.** *Notice that any quadrature rule, such as Newton–Cotes, Gauss–Laguerre, and Gauss–Hermite (see, e.g., [69,70]), used to approximate the integral in* (20), *yields a weighted sum of Gaussian distributions evaluated as a linear function of the values $q_{i}$ and the state $x_{t}$. Furthermore, it is possible to interpret $p(y_{t}|x_{t})$ as a weighted sum of Gaussian distributions in the random variable $x_{t}$. This weighted sum is denoted as the GMM structure. Thus, this structure is considered for developing the Gaussian sum filtering and smoothing algorithms that deal with quantized data.*

**Remark** **3.** *Notice that in [70], a suboptimal filtering algorithm called the quadrature Kalman filter, where a linear regression is used to linearize the nonlinear process and measurement functions using the Gauss–Hermite quadrature rule, was developed. This approach is not directly applicable to the problem of interest in this paper, so that the quantizer is a nondifferentiable nonlinearity.*

*On the other hand, the cubature Kalman filter [70] and smoother [71] are approaches that use the spherical–radial cubature rule to approximate the n-dimensional integrals when computing the expected values in* (10)–(13) *in a nonlinear state-space model. These integral approximations are obtained under the assumption that $p(x_{t}|y_{1:t})$ and $p(x_{t}|y_{1:N})$ are Gaussian distributed. The difference between these approaches and the proposed method in this paper is that the Gauss quadrature rule was used to approximate the integral in the probabilistic model of $p(y_{t}|x_{t})$. It is clear that in this paper, the Gaussian assumption in the filtering and smoothing densities was not used. In fact, it is shown that $p(x_{t}|y_{1:t})$ and $p(x_{t}|y_{1:N})$ are non-Gaussian PDFs.*

### 3.4. Understanding the Gaussian Sum Filtering and Smoothing Algorithms

The general Bayesian framework for filtering leads to the product $p(y_{t}|x_{t})$ and $p(x_{t}|y_{1:t-1})$, as shown in (14). From the definition of $p(y_{t}|x_{t})$ in (21), it is observed that $p(x_{t}|y_{1:t})$ results in the product of two GMMs. This, in turn, results in a GMM with more components than the individual factors $p(y_{t}|x_{t})$ and $p(x_{t}|y_{1:t-1})$. This implies that the time-update equation $p(x_{t+1}|y_{1:t})$ in (15) is also a GMM distribution. A similar situation occurs in the backward-measurement-update equation $p(y_{t:N}|x_{t})$ in (18), which is the product of two GMM structures $p(y_{t}|x_{t})$ and $p(y_{t+1:N}|x_{t})$. This yields another GMM structure with more components than the individual factors, which implies that the backward-prediction equation in (17) is also a GMM structure. For the clarity of the presentation, reducing the product of two GMMs (structures) into one is necessary.

In order to understand the transformation of the product of two summations into another summation, the following sums are considered: $g=\sum_{\tau =1}^{K}g_{\tau }$ and $f=\sum_{\ell =1}^{M}f_{\ell }$. Then, for each two-tuple (τ,ℓ) where τ=1,…,K and ℓ=1,…,M, the product h=fg is another summation and has KM terms indexed by k=(ℓ−1)K+τ. Then, reordering these terms, the following sum is obtained: $h=\sum_{k=1}^{KM}h_{k}$, where $h_{k}=f_{\ell }g_{\tau }$.

### 3.5. Gaussian Sum Filtering for Quantized Data

Using the approximation of the PMF $p(y_{t}|x_{t})$ defined in Theorem 1, the Gaussian sum filtering algorithm can be derived using (14) and (15) as follows:

**Theorem** **2** **(Gaussian sum filter).** *Consider the system in* (4)–(6) *and the approximation of $p(y_{t}|x_{t})$ in* (21). *The filtering equations for state-space systems with quantized output data are defined as follows:*

***Initialization:*** *For t=1, the predicted distribution is $p\left(x_{1}\right)=N_{x_{1}}\left(\mu_{1},P_{1}\right)$, where $p(x_{1})$ is the prior distribution of the initial state. Then, for t=1,…,N, the measurement-update and the time-update equations for the Gaussian sum filtering are defined as follows:*

***Measurement update:*** *The PDF of the current state $x_{t}$ given the current and previous measurements, that is $y_{1},\ldots ,y_{t}$, is the GMM given by:*

(33) $$p\left(x_{t}|y_{1:t}\right)=\sum_{k=1}^{M_{t|t}}\gamma_{t|t}^{k}N_{x_{t}}\left({\hat{x}}_{t|t}^{k},\Sigma_{t|t}^{k}\right),$$

*where $\gamma_{t|t}^{k}$, ${\hat{x}}_{t|t}^{k}$, and $\Sigma_{t|t}^{k}$ are given in Appendix B, $M_{t|t}=KM_{t|t-1}$, and $M_{t|t-1}$ is the number of Gaussian components in the time update step. The initial values satisfy that $M_{1|0}=1$, $\gamma_{1|0}=1$, ${\hat{x}}_{1|0}=\mu_{1}$, and $\Sigma_{1|0}=P_{1}$.*

***Time update:*** *The PDF of the future state $x_{t+1}$, one-step-ahead prediction given the measurements $y_{1},\ldots ,y_{t}$, is the GMM given by:*

(34) $$p\left(x_{t+1}|y_{1:t}\right)=\sum_{k=1}^{M_{t+1|t}}\gamma_{t+1|t}^{k}N_{x_{t+1}}\left({\hat{x}}_{t+1|t}^{k},\Sigma_{t+1|t}^{k}\right),$$

*where $M_{t+1|t}=M_{t|t}$, $\gamma_{t+1|t}^{k}$, ${\hat{x}}_{t+1|t}^{k}$, and $\Sigma_{t+1|t}^{k}$ are given in Appendix B.*

**Proof.**  Consider the recursion in (14) and (15). The required PDF $p(x_{t+1}|x_{t})$ and PMF $p(y_{t}|x_{t})$ are obtained from (5) and (21), respectively. Then, using the measurement-update equations in (14) and Lemma 2 in [65] (p. 86), the following expression is obtained:

(35) $$p\left(x_{t}|y_{1:t}\right)\propto \sum_{\tau =1}^{K}\sum_{\ell =1}^{M_{t|t-1}}\varsigma_{t}^{\tau }\gamma_{t|t-1}^{\ell }N_{\eta_{t}^{\tau }}\left(\kappa_{t}^{\ell \tau },V_{t}^{\ell }\right)N_{x_{t}}\left({\hat{x}}_{t|t}^{k},\Sigma_{t|t}^{k}\right)$$

where $\kappa_{t}^{\ell \tau }$, $V_{t}^{\ell }$, ${\hat{x}}_{t|t}^{k}$, and $\Sigma_{t|t}^{k}$ are defined in (A11), (A12), (A7), and (A8), respectively. Notice that $N_{\eta_{t}^{\tau }}\left(\kappa_{t}^{\ell \tau },V_{t}^{\ell }\right)$ is a coefficient since the measurement $y_{t}$ is available. Then, rewriting the double summation in (35) as a single one with a new index k=(ℓ−1)K+τ, the measurement-update equation in (33) is derived defining $M_{t|t}=KM_{t|t-1}$, the weights ${\bar{\gamma }}_{t|t}^{k}$ as in (A9), and computing the corresponding normalization constant. On the other hand, using the time-update equation in (15) and Lemma 2 in [65] (p. 86), the following equation is obtained:

(36) $$p\left(x_{t+1}|y_{1:t}\right)=\sum_{k=1}^{M_{t|t}}\gamma_{t|t}^{k}\int N_{x_{t}}\left(m_{t}^{k},S_{t}^{k}\right)N_{x_{t+1}}\left({\hat{x}}_{t+1|t}^{k},\Sigma_{t+1|t}^{k}\right)dx_{t},$$

where ${\hat{x}}_{t+1|t}^{k}$ and $\Sigma_{t+1|t}^{k}$ are defined in (A14), and (A15), respectively. Then, solving this integral, the time-update equation in (34) can be derived defining $M_{t+1|t}=M_{t|t}$, the weights $\gamma_{t+1|t}^{k}$ as in (A13), and computing the corresponding normalization constant. This completes the proof.   □

### 3.6. Computing the State Estimator ${\hat{x}}_{t|t}$ from a GMM

Provided $p(x_{t}|y_{1:t})$ in (33) as a GMM, the state estimator given in (10) and the covariance matrix of the estimation error in (12) can be computed as follows:

(37)x^t|t=∑k=1Mt|tγt|tkx^t|tk,(38)Σt|t=∑k=1Mt|tγt|tkΣt|tk+(x^t|tk−x^t|t)(x^t|tk−x^t|t)T.

### 3.7. Backward Filtering for Quantized Data

Using the approximation of the PMF $p(y_{t}|x_{t})$ defined in Theorem 1, the backward filter recursion can be derived as follows:

**Theorem** **3** **(Backward filtering).** *Consider the system in* (4)–(6) *and the approximation of $p(y_{t}|x_{t})$ in* (21). *Then, the backward filter for state-space systems with quantized output data is:*

***Initialization:*** *For t=N, the backward measurement update is given by:*

(39) $$\begin{array}{cc} \; & p\left(y_{N}|x_{N}\right)=\sum_{k=1}^{S_{N|N}}\epsilon_{N|N}^{k}\lambda_{N|N}^{k}\exp\left\{-\frac{1}{2}\left(x_{N}^{T}F_{N|N}^{k}x_{N}-2G_{N|N}^{kT}x_{N}+H_{N|N}^{k}\right)\right\},\; \end{array}$$

*where $S_{N|N}=K$, and: *

(40) $$\begin{array}{cc} \epsilon_{N|N}^{k} & =\varsigma_{N}^{k},\\ F_{N|N}^{k} & =C^{T}R^{-1}C, \end{array}\;\begin{array}{cc} \lambda_{N|N}^{k} & ={\left(\det\left\{2\pi R\right\}\right)}^{-1/2}\\ G_{N|N}^{kT} & =\theta_{N}^{kT}R^{-1}C, \end{array}\;\begin{array}{cc} \theta_{N}^{k} & =\eta_{N}^{k}-Du_{N}-\mu_{N}^{k},\\ H_{N|N}^{k} & =\theta_{N}^{kT}R^{-1}\theta_{N}^{k}, \end{array}$$

*where $\varsigma_{N}^{k}$, $\eta_{N}^{k}$, and $\mu_{N}^{k}$ are defined in Table 1. Then, for t=N−1,…,1, the backward prediction and the backward-measurement-update equations are defined as follows:*

***Backward predictions:*** *The backward-prediction equation is given by:*

(41) $$\begin{array}{cc} \; & p\left(y_{t+1:N}|x_{t}\right)=\sum_{k=1}^{S_{t|t+1}}\epsilon_{t|t+1}^{k}\lambda_{t|t+1}^{k}\exp\left\{-\frac{1}{2}\left(x_{t}^{T}F_{t|t+1}^{k}x_{t}-2G_{t|t+1}^{kT}x_{t}+H_{t|t+1}^{k}\right)\right\}, \end{array}$$

*where $S_{t|t+1}=S_{t+1|t+1}$, $S_{t+1|t+1}$ is the number of components in the backward-measurement-update step, and $\epsilon_{t|t+1}^{k}$, $\lambda_{t|t+1}^{k}$, $F_{t|t+1}^{k}$, $G_{t|t+1}^{kT}$, $H_{t|t+1}^{k}$ are given in Appendix C.*

***Backward-measurement update:*** *The distribution of $y_{t:N}|x_{t}$ evaluated at $y_{t},\ldots ,y_{N}$ is:*

(42) $$\begin{array}{cc} \; & p\left(y_{t:N}|x_{t}\right)=\sum_{k=1}^{S_{t|t}}\epsilon_{t|t}^{k}\lambda_{t|t}^{k}\exp\left\{-\frac{1}{2}\left(x_{t}^{T}F_{t|t}^{k}x_{t}-2G_{t|t}^{kT}x_{t}+H_{t|t}^{k}\right)\right\}, \end{array}$$

*where $S_{t|t}=KS_{t|t+1}$, $S_{t|t+1}$ is the number of components in the backward-prediction step, and $\epsilon_{t|t}^{k}$, $\lambda_{t|t}^{k}$, $F_{t|t}^{k}$, $G_{t|t}^{kT}$, $H_{t|t}^{k}$ are given in Appendix C.*

**Proof.**  Consider the recursion in (17) and (18). The required PDF $p(x_{t+1}|x_{t})$ and PMF $p(y_{t}|x_{t})$ are given by Equations (5) and (21), respectively. The proof is carried out by induction in reverse time. First, it is verified that it holds for t=N−1, then it is assumed to be true for t=s+1, and finally, it is verified that it holds for t=s. Notice that the recursion starts in t=N with $p\left(y_{N:N}|x_{N}\right)=p\left(y_{N}|x_{N}\right)$, which is defined in (39). From (17), at time t=N−1, the backward-prediction step is defined as:

(43) $$p\left(y_{N:N}|x_{N-1}\right)=\int p\left(y_{N:N}|x_{N}\right)p\left(x_{N}|x_{N-1}\right)dx_{N},$$

where $p(y_{N:N}|x_{N})$ is given by (39) and $p(x_{N}|x_{N-1})$ is defined by the system model in (5). From the definition given in (43), the equation below is obtained:

(44) $$\begin{array}{c} p\left(y_{N:N}|x_{N-1}\right)=\sum_{k=1}^{S_{N|N}}\frac{\epsilon_{N|N}^{k}\lambda_{N|N}^{k}}{\sqrt{\det\left\{2\pi Q\right\}}}\int \exp\left\{-\frac{1}{2}\left[x_{N}^{T}{\bar{P}}_{N}^{k}x_{N}-2{\bar{V}}_{N}^{kT}x_{N}+{\bar{S}}_{N}^{k}\right]\right\}dx_{N}, \end{array}$$

where ${\bar{P}}_{N}^{k}=\left(F_{N|N}^{k}+Q^{-1}\right)$, ${\bar{V}}_{N}^{k}=\left(G_{N|N}^{k}+J_{N-1}\right)$, ${\bar{S}}_{N}^{k}=\left(H_{N|N}^{k}+L_{N-1}\right)$, $J_{N-1}^{T}={\left(Ax_{N-1}+Bu_{N-1}\right)}^{T}Q^{-1}$, and $L_{N-1}={\left(Ax_{N-1}+Bu_{N-1}\right)}^{T}Q^{-1}\left(Ax_{N-1}+Bu_{N-1}\right)$. Then, completing the square and solving the integral in (44), it is obtained:

(45) $$\begin{array}{cc} p(y_{N:N}|x_{N-1}) & =\sum_{k=1}^{S_{N-1|N}}\epsilon_{N-1|N}^{k}\lambda_{N-1|N}^{k}\\ & \times \exp\left\{-\frac{1}{2}\left(x_{N-1}^{T}F_{N-1|N}^{k}x_{N-1}-2G_{N-1|N}^{kT}x_{N-1}+H_{N-1|N}^{k}\right)\right\}, \end{array}$$

where $S_{N-1|N}=S_{N|N}$ and the remaining terms in (45) are defined in (A22)–(A26), but evaluated at t=N−1. The backward-measurement-update step in (18) is as follows:

(46) $$p\left(y_{N-1:N}|x_{N-1}\right)=p\left(y_{N-1}|x_{N-1}\right)p\left(y_{N:N}|x_{N-1}\right).$$

Thus, using (45) in the definition given in (46), it is obtained:

(47) $$\begin{array}{cc} p(y_{N-1:N}|x_{N-1}) & =\sum_{\tau =1}^{K}\sum_{\ell =1}^{S_{N-1|N}}\frac{\varsigma_{N-1}^{\tau }\epsilon_{N-1|N}^{\ell }\lambda_{N-1|N}^{\ell }}{\sqrt{\det\left\{2\pi R\right\}}}\\ \; & \times \exp\left\{-\frac{1}{2}\left(x_{N-1}^{T}{\tilde{F}}_{N-1}x_{N-1}-2{\tilde{G}}_{N-1}^{\tau T}x_{N-1}+{\tilde{H}}_{N-1}^{\tau }\right)\right\}\\ \; & \times \exp\left\{-\frac{1}{2}\left(x_{N-1}^{T}F_{N-1|N}^{\ell }x_{N-1}-2G_{N-1|N}^{\ell T}x_{N-1}+H_{N-1|N}^{\ell }\right)\right\}, \end{array}$$

where ${\tilde{F}}_{N-1}=C^{T}R^{-1}C$, ${\tilde{G}}_{N-1}^{\tau T}=\theta_{N-1}^{\tau T}R^{-1}C$, and ${\tilde{H}}_{N-1}^{\tau }=\theta_{N-1}^{\tau T}R^{-1}\theta_{N-1}^{\tau }$ with $\theta_{N-1}^{\tau }=\eta_{N-1}^{\tau }-Du_{N-1}-\mu_{N-1}^{\tau }$. Finally, rewriting the double summation in the last equation into a single one with a new index k=(ℓ−1)K+τ results in:

(48) $$\begin{array}{cc} \; & p\left(y_{N-1:N}|x_{N-1}\right)=\sum_{k=1}^{S_{N-1|N-1}}\epsilon_{N-1|N-1}^{k}\lambda_{N-1|N-1}^{k}\\ \; & \;\;\times \exp\left\{-\frac{1}{2}\left[x_{N-1}^{T}F_{N-1|N-1}^{k}x_{N-1}-2G_{N-1|N-1}^{kT}x_{N-1}+H_{N-1|N-1}^{k}\right]\right\}, \end{array}$$

where $S_{N-1|N-1}=KS_{N-1|N}$ and the remaining terms in (48) are the same as the ones shown in (A16)–(A21), but evaluated at t=N−1. Thus, it is concluded that it holds for t=N−1. A similar procedure was applied to find that, for both the backward-prediction and backward-measurement-update steps in the backward filter, it yields the same expressions in (A22)–(A26) and (A16)–(A21), but evaluated at t=s. Thus, it is concluded that Theorem 3 holds for all *t*.    

From Theorems 2 and 3, it is clear that the number of elements in the mixture grows exponentially with every iteration, making the algorithm computationally intractable after a few iterations. In addition, it would be necessary to save and manage a large amount of information in every iteration of the Gaussian sum filtering and in the backward recursion. Therefore, an algorithm that reduces the number of GMM components should be implemented in every iteration of these two algorithms in order to keep the number of components bounded. Different methods have been proposed to perform this kind of procedure, termed Gaussian sum reduction, such us pruning, joining, and integral-squared-error-based methods; see [70]. In this work, the Kullback–Leibler approach for Gaussian sum reduction proposed in [64,72] was used. The idea behind the Gaussian sum reduction is to transform the GMM ${\left\{\varphi_{i},\upsilon_{i},\Gamma_{i}\right\}}_{i=1}^{J}$ into a GMM ${\left\{\varphi_{i},\upsilon_{i},\Gamma_{i}\right\}}_{i=1}^{S}$, where 1≤S≤J. In [64], it was suggested to use a measure of dissimilarity between two components and pooling the pair of components that minimize this measure. Then, based on this idea, in [72], the Kullback–Leibler information number was used as the measure of dissimilarity. The author in [72] provided an algorithm to merge two components so that the merged component preserves the first- and second-order moments of the original two components, which is given by:

(49) $$\left(\varphi_{ij},\upsilon_{ij},\Gamma_{ij}\right)=M\left\{\left(\varphi_{i},\upsilon_{i},\Gamma_{i}\right),\left(\varphi_{j},\upsilon_{j},\Gamma_{j}\right)\right\},$$

where $M\left\{\cdot ,\cdot \right\}$ is a merging function such that:

(50)φij=φi+φj,(51)υij=φ¯iυi+φ¯jυj,(52)Γij=φ¯iΓi+φ¯jΓj+φ¯iφ¯jυi−υjυi−υjT,

where ${\bar{\varphi }}_{i}=\varphi_{i}/\left(\varphi_{i}+\varphi_{j}\right)$ and ${\bar{\varphi }}_{j}=\varphi_{j}/\left(\varphi_{i}+\varphi_{j}\right)$. The merging function applied to two components *i* and *j* (i≠j) minimizes the dissimilarity measure D(i,j), defined as:

(53) $$\begin{array}{c} D\left(i,j\right)=\frac{1}{2}\left[\varphi_{ij}\log\det\left\{\Gamma_{ij}\right\}-\varphi_{i}\log\det\left\{\Gamma_{i}\right\}-\varphi_{j}\log\det\left\{\Gamma_{j}\right\}\right], \end{array}$$

The function D(i,j) satisfies D(i,j)=D(j,i) and D(i,i)=0. This implies that the total number of combinations to merge is reduced to 0.5J(J−1). The authors in [73] used Runnalls’ algorithm in a Bayesian filtering environment, and they modified it to include a user-defined threshold for the number of components after reduction and a user-defined threshold ε that satisfies D(i,j)<ε. In Algorithm 1, the steps for implementing the Gaussian sum filtering are summarized.
**Algorithm 1** Gaussian sum filter algorithm for quantized output data_  **1**_ **Input:** The PDF of the initial state $p(x_{1})$, e.g., $M_{1|0}=1$, $\gamma_{1|0}=1$, ${\hat{x}}_{1|0}=\mu_{1}$,
     $\Sigma_{1|0}=P_{1}$. The points of the Gauss–Legendre quadrature ${\left\{\omega_{\tau },\psi_{\tau }\right\}}_{\tau =1}^{K}$.

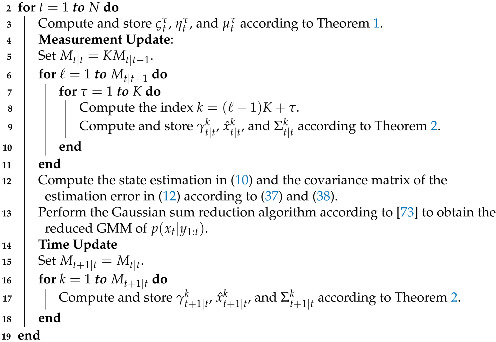
 _**20**_ **Output:** The state estimation in (10), the covariance matrix of the estimation error   in (12), the filtering PDFs $p(x_{t}|y_{1:t})$, the predictive PDFs $p(x_{t+1}|y_{1:t})$, and the set   $\left\{\varsigma_{t}^{\tau },\eta_{t}^{\tau },\mu_{t}^{\tau }\right\}$, for t=1,…,N.

The backward recursion in Theorem 3 is used to obtain the smoothing PDF in (19). For this purpose, $p(y_{t:N}|x_{t})$ is converted into a GMM structure of the random variable $x_{t}$. Then, the Gaussian sum reduction algorithm is applied to the GMM structure of $p(y_{t:N}|x_{t})$ to obtain:

(54) $$p\left(y_{t:N}|x_{t}\right)=\sum_{k=1}^{S_{\mathrm{red}}}\delta_{t|t}^{k}N_{x_{t}}\left(z_{t|t}^{k},U_{t|t}^{k}\right),$$

where $S_{\mathrm{red}}$ is the number of Gaussian components kept after the Gaussian reduction procedure and $\delta_{t|t}^{k}$, $z_{t|t}^{k}$, and $U_{t|t}^{k}$ are the corresponding weight, mean, and covariance matrix. This reduced GMM structure is used to obtain the smoothing PDFs. However, for the next recursion in the backward filter, reconverting the reduced GMM structure into the backward filter form to obtain the reduced version of the backward-measurement-update step in (42) is required. This conversion process between the backward filter and GMM structure is summarized in Lemma A3 in Appendix A. In Algorithm 2, the steps for implementing the backward filter are summarized.

**Algorithm 2** Backward-filtering algorithm for quantized output data  _1_  **Input:** The initial backward measurement $p(y_{N}|x_{N})$ given in (39), e.g., $S_{N|N}$, $\epsilon_{N|N}^{k}$,
     $\lambda_{N|N}^{k}$, $F_{N|N}^{k}$, $G_{N|N}^{kT}$, and $H_{N|N}^{k}$. The set $\left\{\varsigma_{t}^{\tau },\eta_{t}^{\tau },\mu_{t}^{\tau }\right\}$ for t=1,…,N computed in Algorithm 1.


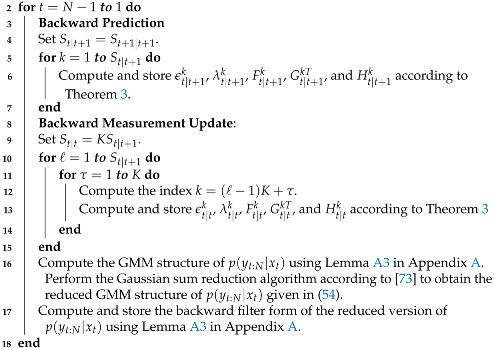

 _**19**_  **Output:** The backward prediction $p(y_{t+1:N}|x_{t})$ and the backward measurement    update $p(y_{t:N}|x_{t})$ for t=N,…,1.

### 3.8. Smoothing Algorithm with Quantized Data

In order to obtain the smoothing PDF in (19), the GMM structure $p(y_{t:N}|x_{t})$ in the random variable $x_{t}$ given in (54) is used. This GMM structure is multiplied by the time-update equation $p(x_{t}|y_{1:t-1})$ of the filtering algorithm given in (34). Then, the smoothing PDF is obtained from the following:

**Theorem** **4** **(Gaussian sum smoothing).** *Consider the system in* (4)–(6). *Given $p(x_{t}|y_{1:t-1})$, $p(x_{N}|y_{1:N})$ and $p(y_{t:N}|x_{t})$, then the smoothing PDF at time t=N is given by $p(x_{N}|y_{1:N})$, and for t=N−1,…,1, the PDF $p(x_{t}|y_{1:N})$ is a GMM given by:*

(55) $$p\left(x_{t}|y_{1:N}\right)=\sum_{k=1}^{S_{t|N}}\epsilon_{t|N}^{k}N_{x_{t}}\left({\hat{x}}_{t|N}^{k},\Sigma_{t|N}^{k}\right),$$

*where $S_{t|N}=M_{t|t-1}S_{red}$, $M_{t|t-1}$ and $S_{red}$ are given in* (34) and (54), *respectively, and $\epsilon_{t|N}^{k}$, ${\hat{x}}_{t|N}^{k}$, and $\Sigma_{t|N}^{k}$ are given in Appendix D.*

**Proof.**  Consider the definition of Bayesian smoothing given in (19), the time-update equation $p(x_{t}|y_{1:t-1})$ obtained from (34), and the reduced version of the measurement-update step in the backward filter $p(y_{t:N}|x_{t})$ defined in (54). For t=N−1,N−2,…,1, it is obtained:

(56) $$p\left(x_{t}|y_{1:N}\right)\propto \sum_{\tau =1}^{M_{t|t-1}}\sum_{\ell =1}^{S_{\mathrm{red}}}\gamma_{t|t-1}^{\tau }\delta_{t|t}^{\ell }N_{x_{t}}\left(z_{t|t}^{\ell },U_{t|t}^{\ell }\right)N_{x_{t}}\left({\hat{x}}_{t|t-1}^{\tau },\Sigma_{t|t-1}^{\tau }\right).$$

Defining $g\left(x_{t}\right)=N_{x_{t}}\left(z_{t|t}^{\ell },U_{t|t}^{\ell }\right)N_{x_{t}}\left({\hat{x}}_{t|t-1}^{\tau },\Sigma_{t|t-1}^{\tau }\right)$, the following equation is derived:

(57) $$\begin{array}{c} g\left(x_{t}\right)=\frac{\exp\left\{-\frac{1}{2}\left[\phi_{1t}^{\ell }+\phi_{2t}^{\tau }-\phi_{3t}^{\ell \tau }\right]\right\}N_{x_{t}}\left({\left(L_{t}^{\ell \tau }\right)}^{-1}\rho_{t}^{\ell \tau },{\left(L_{t}^{\ell \tau }\right)}^{-1}\right)}{{\left(2\pi \right)}^{\frac{n}{2}}\sqrt{\det\left\{L_{t}^{\ell \tau }\right\}\det\left\{U_{t|t}^{\ell }\right\}\det\left\{\Sigma_{t|t-1}^{\tau }\right\}}}, \end{array}$$

where $L_{t}^{\ell \tau }$, $\rho_{t}^{\ell \tau }$, $\phi_{1t}^{\ell }$, $\phi_{2t}^{\tau }$, and $\phi_{3t}^{\ell \tau }$ are defined in (A32), (A31), (A34), (A35), and (A33), respectively. Next, expressing the double summation in (56) as a single one with a new index $k=\left(\ell -1\right)M_{t|t-1}+\tau$, it is obtained:

(58) $$\begin{array}{c} p\left(x_{t}|y_{1:N}\right)\propto \sum_{k=1}^{S_{t|N}}{\bar{\epsilon }}_{t|N}^{\;k}N_{x_{t}}\left({\hat{x}}_{t|N}^{k},\Sigma_{t|N}^{k}\right), \end{array}$$

where $S_{t|N}=M_{t|t-1}S_{\mathrm{red}}$ and ${\bar{\epsilon }}_{t|N}^{\;k}$, $\Sigma_{t|N}^{k}$, and ${\hat{x}}_{t|N}^{k}$ are given in (A30), (A28), and (A29), respectively. Finally, the smoothing PDF in (55) is obtained by computing the normalized wights as (A27), and this completes the proof.   □

Provided $p(x_{t}|y_{1:N})$ in (55) as a GMM, the state estimator given in (11) and the covariance matrix of the estimation error in (13) can be computed as follows:

(59)x^t|N=∑k=1St|Nϵt|Nkx^t|Nk,(60)Σt|N=∑k=1St|Nϵt|NkΣt|Nk+(x^t|Nk−x^t|N)(x^t|Nk−x^t|N)T.

In Algorithm 3, the steps to implement the Gaussian sum smoothing are summarized.

**Algorithm 3** Gaussian sum smoothing algorithm for quantized output data_  **1**_  **Input:** The PDFs $p(x_{t}|y_{1:t-1})$ and $p(x_{N}|y_{1:N})$ obtained from Algorithm 1 and
     $p(y_{t:N}|x_{t})$ obtained from Algorithm 2._  **2**_  Save the PDF $p(x_{N}|y_{1:N})$

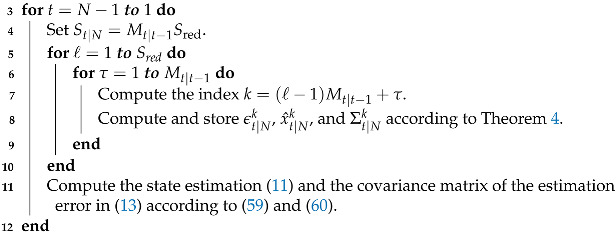

 _**13**_  **Output:** The state estimation in (11), the covariance matrix of the estimation error     in (13), and the smoothing PDFs $p(x_{t}|y_{1:N})$, for t=1,…,N.

### 3.9. Computing the Smoothing Joint PDF $p(x_{t+1},x_{t}|y_{1:N})$

In Theorems 2–4, the filtering and smoothing problems for quantized data are solved. However, many strategies for the identification of state-state space systems [8,9] require the joint PDF $p(x_{t+1},x_{t}|y_{1:N})$, which for the quantized output data case is given by the following:

**Theorem** **5.** *Consider the system in* (4)–(6), *the PDF $p(x_{t}|y_{1:t})$ and the backward prediction equation $p(y_{t+1:N}|x_{t+1})$ given in Theorems 2 and 3, respectively, and the PDF $p(x_{t+1}|x_{t})$ given in Equation* (5). *Then, for t=N−1,…,1, the joint PDF $p(x_{t+1},x_{t}|y_{1:N})$ is the GMM given by:*

(61) $$p\left(x_{t+1},x_{t}|y_{1:N}\right)=\sum_{k=1}^{S_{t,t+1}}\alpha_{k}N_{\chi_{t}}\left({\hat{\chi }}_{t|N}^{k},E_{t|N}^{k}\right),$$

*where $S_{t,t+1}=M_{t|t}S_{t+1|t+1}$, $M_{t|t}$ and $S_{t+1|t+1}$ are given in* (33) *and* (42), *respectively, $\alpha_{k}$, ${\hat{\chi }}_{t|N}^{k}$, and $E_{t|N}^{k}$ are given in Appendix E, and $\chi_{t}$ is the extended vector given by:*

(62) $$\chi_{t}^{T}={\left[\begin{array}{cc} x_{t+1}^{T} & x_{t}^{T} \end{array}\right]}^{T}.$$

*At time t=N, the joint PDF $p(x_{N+1},x_{N}|y_{1:N})$ is given by* (61) *with:*

(63) $$\begin{array}{cc} \; & S_{N+1|N+1}=1,\;\epsilon_{N+1|N+1}^{\ell }=1,\;\lambda_{N+1|N+1}^{\ell }=1,\\ \; & F_{N+1|N+1}^{\ell }=0,\;G_{N+1|N+1}^{\ell T}=0,\;H_{N+1|N+1}^{\ell }=0. \end{array}$$

**Proof.**  Using Bayes’ theorem, the joint PDF $p(x_{t+1},x_{t}|y_{1:N})$ can be obtained as:

(64) $$p\left(x_{t+1},x_{t}|y_{1:N}\right)=\frac{p\left(y_{t+1:N}|x_{t+1}\right)p\left(x_{t+1}|x_{t}\right)p\left(x_{t}|y_{1:t}\right)}{p(y_{t+1:N}|y_{1:t})},$$

where $p(y_{t+1:N}|y_{1:t})$ is a normalization constant. The required PDFs $p(x_{t}|y_{1:t})$ and $p(x_{t+1}|x_{t})$ and backward-prediction equation $p(y_{t+1:N}|x_{t+1})$ are given in (33), (5) and (42), respectively. Using Lemma A2, $p(x_{t}|y_{1:t})$ in (33) can be rewritten as:

(65) $$\begin{array}{c} p\left(x_{t}|y_{1:t}\right)=\sum_{\tau =1}^{M_{t|t}}\frac{\gamma_{t|t}^{\tau }}{\sqrt{\det\left\{2\pi \Sigma_{t|t}^{\tau }\right\}}}\exp\left\{-\frac{1}{2}\left(x_{t}^{T}{\left(\Sigma_{t|t}^{\tau }\right)}^{-1}x_{t}-2J_{t}^{\tau T}x_{t}+L_{t}^{\tau }\right)\right\}, \end{array}$$

where $J_{t}^{\tau T}$ and $L_{t}^{\tau }$ are defined in (A44) and (A45), respectively. Considering $p(y_{t+1:N}|x_{t+1})$, $p(x_{t}|y_{1:t})$, $p(x_{t+1}|x_{t})$, and the extended vector of the state given in (62), the argument of these three functions can be written as follows:

(66) $$A_{1}=\chi_{t}^{T}\left[\begin{array}{cc} 0 & 0\\ 0 & {\left(\Sigma_{t|t}^{\tau }\right)}^{-1} \end{array}\right]\chi_{t}-2\left[\begin{array}{cc} 0 & J_{t}^{\tau T} \end{array}\right]\chi_{t}+L_{t}^{\tau },$$

(67) $$A_{2}=\chi_{t}^{T}\left[\begin{array}{cc} F_{t+1|t+1}^{\ell } & 0\\ 0 & 0 \end{array}\right]\chi_{t}-2\left[\begin{array}{cc} G_{t+1|t+1}^{\ell T} & 0 \end{array}\right]\chi_{t}+H_{t+1|t+1}^{\ell },$$

(68) $$\begin{array}{cc} A_{3} & =\chi_{t}^{T}\left[\begin{array}{cc} Q^{-1} & -Q^{-1}A\\ -A^{T}Q^{-1} & A^{T}Q^{-1}A \end{array}\right]\chi_{t}\\ & -2\left[\begin{array}{cc} u_{t}^{T}B^{T}Q^{-1} & -u_{t}^{T}B^{T}Q^{-1}A \end{array}\right]\chi_{t}+u_{t}^{T}B^{T}Q^{-1}Bu_{t}, \end{array}$$

Then, adding these expressions, the following equation is derived:

(69) $$\begin{array}{cc} p(x_{t+1},x_{t}|y_{1:N}) & \propto \sum_{\tau =1}^{M_{t|t}}\sum_{\ell =1}^{S_{t+1|t+1}}\frac{\gamma_{t|t}^{\tau }\epsilon_{t+1|t+1}^{\ell }\lambda_{t+1|t+1}^{\ell }}{{\left(2\pi \right)}^{n}\sqrt{\det\left\{Q\right\}\det\left\{\Sigma_{t|t}^{\tau }\right\}}}\\ \; & \times \exp\left\{-\frac{1}{2}\left(\chi_{t}^{T}F_{t}^{\ell \tau }\chi_{t}-2G_{t}^{\ell \tau T}\chi_{t}+H_{t}^{\ell \tau }\right)\right\}, \end{array}$$

where $F_{t}^{\ell \tau }$, $G_{t}^{\ell \tau T}$, and $H_{t}^{\ell \tau }$ are defined in (A41), (A42), and (A43), respectively. Completing the square and expressing the double summation as a single one with a new index $k=\left(\ell -1\right)M_{t|t}+\tau$, it is obtained:

(70) $$\begin{array}{c} p\left(x_{t+1},x_{t}|y_{1:N}\right)\propto \sum_{k=1}^{S_{t,t+1}}{\bar{\alpha }}_{k}N_{\chi_{t}}\left({\hat{\chi }}_{t|N}^{k},E_{t|N}^{k}\right), \end{array}$$

where $S_{t,t+1}=M_{t|t}S_{t+1|t+1}$, ${\bar{\alpha }}_{k}$ is defined in (A39), and ${\hat{\chi }}_{t|N}^{k}$ and $E_{t|N}^{k}$ are defined in (A37) and (A38), respectively. Finally, the smoothing joint distribution in (64) for t=N−1,…,1 is derived by computing the normalized wights as in (A36). Notice that, for t=N, the smoothing joint PDF $p\left(x_{N+1},x_{N}|y_{1:N}\right)=p\left(x_{N+1}|x_{N}\right)p\left(x_{N}|y_{1:N}\right)$ is obtained as (61) considering the expressions given in (63). Then, the proof is finished.   □

In Algorithm 4, the steps to implement the Gaussian sum smoothing for computing the joint PDF $p(x_{t+1},x_{t}|y_{1:N})$ are summarized.
**Algorithm 4** Gaussian sum smoother to compute $p(x_{t+1},x_{t}|y_{1:N})$ for quantized output data_  **1**_  **Input:** The PDF $p(x_{t}|y_{1:t})$ obtained in Algorithm 1, $p(y_{t+1:N}|x_{t+1})$ computed in     Algorithm 2, and the PDF $p(x_{t+1}|x_{t})$ given in Equation (5).
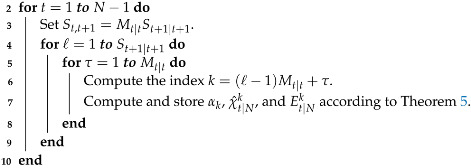
 _**11**_  Compute and store $p(x_{N+1},x_{N}|y_{1:N})$ according to Theorem 5 with $S_{N+1|N+1}$,    $\epsilon_{N+1|N+1}^{\ell }$, $\lambda_{N+1|N+1}^{\ell }$, $F_{N+1|N+1}^{\ell }$, $G_{N+1|N+1}^{\ell T}$, and $H_{N+1|N+1}^{\ell }$ given in (63). _**12**_  **Output:** The smoothing PDFs $p(x_{t+1},x_{t}|y_{1:N})$, for t=1,…,N.

## 4. Numerical Example

In this section, a numerical example to illustrate the benefits of this paper proposal is presented. Furthermore, a practical simulation example is studied: the problem of estimating the tank liquid level in a two-tank system is addressed. Typically, a numerical simulation approach is used for testing new algorithms and designs in applications of state estimation [74], control [75], and system identification [63], among others. This approach is used to evaluate the performance of the estimation algorithms, in order to avoid safety problems that can occur in a real-world processes.

To evaluate the performance of the proposed filtering and smoothing methods for quantized data, a comparison with three classical techniques is presented: standard Kalman filtering and smoothing [76], quantized Kalman filtering and smoothing [49,77], and particle filtering and smoothing [31]. Notice that it is also possible to implement a version of particle filtering and smoothing algorithms using the approximation of $p(y_{t}|x_{t})$ given in (21). However, in the numerical examples run for filtering, the computation time was high. In order to validate the approximation in Theorem 1, the standard particle filtering and smoothing algorithms with a large number of particles were used, where $p(y_{t}|x_{t})$ was computed using the integral in (20) from the MATLAB function *mvncdf*, which computes the multivariate normal cumulative distribution function. The *true* filtering and smoothing PDFs were considered to be provided by the particle filter and smoother with 20,000 particles, which was defined as the ground truth.

In the following examples, the discrete-time system in state-space form given in (1)–(3) is used with:

(71) $$y_{t}=\Delta_{q}\mathrm{round}\left\{z_{t}/\Delta_{q}\right\},$$

where the quantizer is defined in terms of the *round* function in MATLAB and the quantization step $\Delta_{q}$. The infimum and supremum values of the sets $J_{i}$ defined in (8), $q_{i-1}$ and $q_{i}$, can be calculated for the infinite-level quantizer as $q_{i-1}=y_{t}-0.5\Delta_{q}$ and $q_{i}=y_{t}+0.5\Delta_{q}$ for i=…,1,2…,L,….

The experiments were carried out on a computer with the following specifications: processor: Intel(R) Core(TM) i5-8300H CPU @ 2.30GHz, RAM memory: 8.00 GB, operating system: Windows 10 with MATLAB 2020b.

### 4.1. Example 1: First-Order System

**Figure 5:**
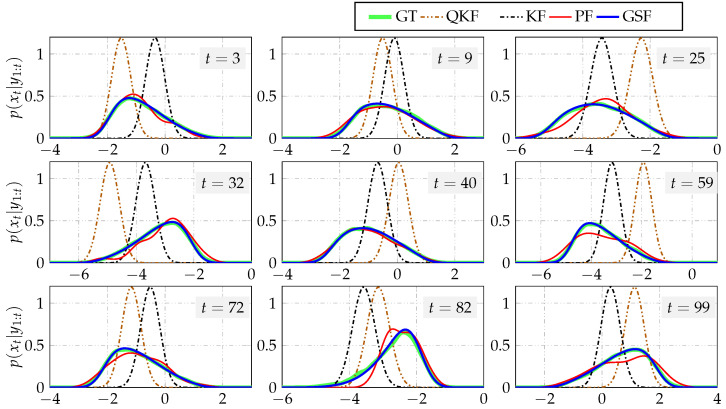
Example 1. Filtering PDFs at some instants of time for $\Delta_{q}=7$, K=10, and 100 particles. GT stands for ground truth. QKF, KF, PF, and GSF stand for quantized Kalman filter, Kalman filter, particle filter, and Gaussian sum filter, respectively.

In this example, the following state-space system was considered:

(72)xt+1=0.9xt+1.0ut+wt,(73)zt=2.0xt+0.5ut+vt,

where $w_{t}∼N_{w_{t}}\left(0,1\right)$ and $v_{t}∼N_{v_{t}}\left(0,0.5\right)$. Furthermore, the input was considered to be drawn from $N_{u_{t}}\left(0,2\right)$ and $x_{1}∼N_{x_{1}}\left(1,1\right)$. In Figure 5, the filtering PDFs for some time instants are shown. The quantization step used in this example and the number of Gaussian components to approximate $p(y_{t}|x_{t})$ were considered to be $\Delta_{q}=7$ and K=10, respectively. Furthermore, $S_{\mathrm{red}}=K$.

Figure 5 shows that the filtering PDFs obtained with our proposal, the Gaussian sum filter, were that best fit to the ground truth. In contrast, the PDFs obtained with the Kalman filter, quantized Kalman filter, and particle filter were different from the ground truth. Notice that the results obtained using the particle filter with 100 particles were good at t=3,9,40, whilst the resulting PDFs for t=25,32,59,72,82,99 differed slightly more from the ground truth. However, the performance of particle filtering can be improved by increasing the number of particles, which produces an increment in the execution time.

**Figure 6:**
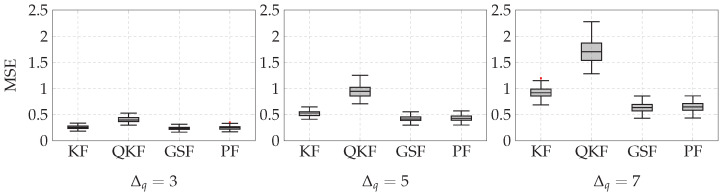
MSE between the true and estimated state for $\Delta_{q}=\left\{3,5,7\right\}$. KF, QKF, GSF, and PF stand for the Kalman filter, quantized Kalman filter, Gaussian sum filter, and particle filter, respectively.

To compare the accuracy of the state estimation, 100 Monte Carlo trials were run, and the mean-squared error between the true state and the estimation obtained by the Kalman filter, quantized Kalman filter, Gaussian sum filter, and particle filter is computed as follows:

(74) $$\mathrm{MSE}=\frac{1}{R}\sum_{k=1}^{R}\|x_{t}-{\hat{x}}_{t|t}{\|}_{2}^{2},$$

where $x_{t}$ is the true state (which in a real-word system is not available, but in simulations, it can be used to analyze the performance of the estimation techniques), ${\hat{x}}_{t|t}$ is the estimation of the state system, and $\|\cdot {\|}_{2}^{2}$ denotes the squared Euclidean norm. In Figure 6, the box plots corresponding to 100 Monte Carlo runs for $\Delta_{q}=\left\{3,5,7\right\}$ are shown.

Figure 6 shows that the MSE between the true state and the estimation obtained with the Kalman filter and quantized Kalman filter increased fast as $\Delta_{q}$ increased. However, the MSE increased slowly for the state estimation obtained with the Gaussian sum filter and particle filter.

**Figure 7:**
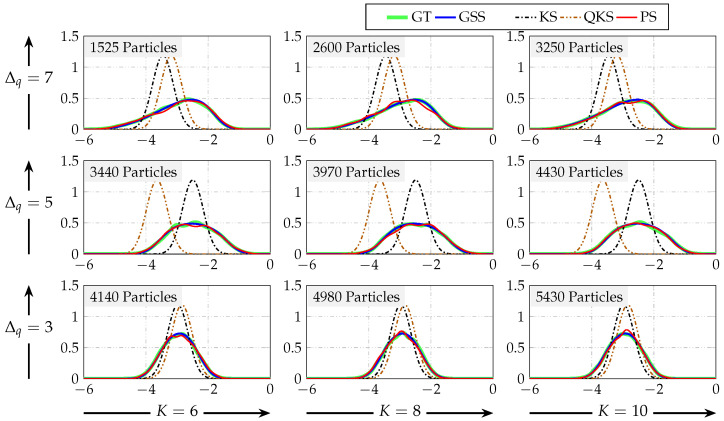
Smoothing PDF at time t=100, for $K=\left\{6,8,10\right\}$, where *K* increases to the right, and for $\Delta_{q}=\left\{3,5,7\right\}$, where $\Delta_{q}$ increases upwards. GT stands for the ground truth. GSS, KS, QKS, and PS stand for the Gaussian sum smoother, Kalman smoother, quantized Kalman smoother, and particle smoother, respectively.

In Figure 7, the smoothing PDFs at time t=100 are shown. The quantization step was considered to be chosen from $\Delta_{q}=\left\{3,5,7\right\}$, and the number of Gaussian components to approximate $p(y_{t}|x_{t})$ was chosen from $K=\left\{6,8,10\right\}$. Furthermore, the number of Gaussian components kept after the reduction procedure was considered as $S_{\mathrm{red}}=K$. In order to obtain the adequate number of particles to compare the particle smoother with the Gaussian sum smoother, 100 Monte Carlo simulations were carried out to obtain the number of particles that yielded smoothing PDFs that were as close to the true PDFs as the Gaussian sum smoother using $K=\left\{6,8,10\right\}$. For comparison purposes, the particle smoother execution time corresponding to the time required to implement the particle smoother algorithm using the number of particles that produces a similar result to the Gaussian sum smoother with *K* components is defined as *Par(K)*. The $L_{2}$-norm of the difference between the true and the estimated PDFs as the measure of similarity was used:

(75) $$\|q-\hat{q}{\|}_{2}={\left[\sum_{k=1}^{M}{\left|q-\hat{q}\right|}^{2}\right]}^{1/2},$$

where *q* represents the true PDF and $\hat{q}$ represents the estimated PDF, which was chosen so that $\|q-\hat{q}{\|}_{2}<1\times 10^{-6}$. The approximated number of particles (labeled in each PDF in Figure 7) was used to compare the execution time of both algorithms, the Gaussian sum smoother and particle smoother, and the results are shown in Table 2. Figure 7 shows that the smoothing PDFs obtained with our proposal using a small number of Gaussian components and the PDFs obtained using the particle smoother with a large number of particles fit the ground truth. In contrast, the PDFs obtained with the Kalman smoother and quantized Kalman smoother were different from the ground truth. Furthermore, the execution time in Table 2 shows that the required time to perform the Gaussian sum smoother was less compared to the time to perform the particle smoother, which needs a large number of particles to produce a result similar to the Gaussian sum smoother.

From the results shown in Figure 5, Figure 6 and Figure 7 and Table 2, it can be concluded that:

- The filtering and smoothing PDFs are non-Gaussian, although the process and output noises in (1) and (2) are Gaussian distributed;
- The accuracy of the standard and quantized Kalman filtering and smoothing decreased as the quantization step increased;
- The state estimates obtained with particle filter and smoother were similar to the results obtained using the Gaussian sum filter and smoother. However, the characterization of the filtering and smoothing PDFs using the Gaussian sum filter and smoother were better than the PDF obtained by the particle filter and smoother. Notice that a correct characterization of a PDF is important when high-order moments need to be computed, especially in system identification tasks;
- In order to implement the Gaussian sum filter and smoother, the parameters *K* (the number that defines the quality of the $p(y_{t}|x_{t})$ approximation) and $S_{\mathrm{red}}$ (the Gaussian components kept after the Gaussian sum reduction algorithm) need to be chosen by the user. These parameters can be found in a simulation study by the trial and error approach and should be set by a trade-off between the time complexity and the accuracy of the estimation. A large value of *K* produces an accurate estimate, but a high computational load;
- The larger the quantization step is, the larger the number of Gaussian components, *K*, needed to approximate $p(y_{t}|x_{t})$ in order to obtain an accurate approximation. However, for a large quantization step, the number *K* needed to obtain a good approximation of the filtering and smoothing PDFs is relatively small compared to the number of particles required to obtain similar results using the particle filter and smoother;
- The maximum number of Gaussian components kept after the Gaussian reduction procedure is important for the accuracy of the approximation. In the simulations, $S_{\mathrm{red}}=K$ was used. Furthermore, it was noticed that once an adequate $S_{\mathrm{red}}$ was defined, incrementing this value did not produce a significant improvement in the estimation. However, this increment in $S_{\mathrm{red}}$ was really critical for the resulting numerical complexity of the algorithm (and hence, the execution time), which increased since the Gaussian sum reduction procedure (e.g., Kullback–Leibler reduction) utilized more time to reduce a large amount of Gaussian components;
- The Gaussian sum smoother execution time for all values of $\Delta_{q}$ was small. This occurred because in each case, a relatively small number of Gaussian components to approximate $p(y_{t}|x_{t})$ were used. However, the particle smoother execution time is variable for different values of $\Delta_{q}$. As $\Delta_{q}$ decreased, the $L_{2}$-norm between the Gaussian sum smoother and the ground truth decreased, and a larger number of particles to obtain a comparable $L_{2}$-norm between the particle smoother and the ground truth were required.

### 4.2. Real-World Application: Tank Liquid Level

**Figure 8:**
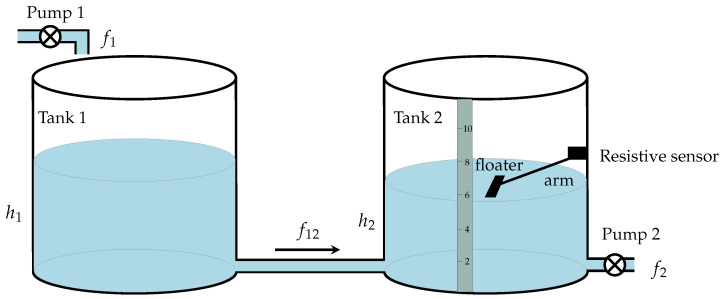
Two-tank system. $h_{1}$ and $h_{2}$ denote the liquid level in Tank 1 and Tank 2, respectively. The liquid flows into Tank 1 at a rate $f_{1}$ and out of Tank 2 at a rate $f_{2}$. The quantizer has minimum and maximum values $\beta_{1}=2$ and $\beta_{L}=10$.

In this example, the problem of estimating the tank liquid level in a two-tank system by using the measurements taken by a low-cost sensor based on a variable resistor that is attached to an arm with a floater is considered; see Figure 8.

**Figure 9:**
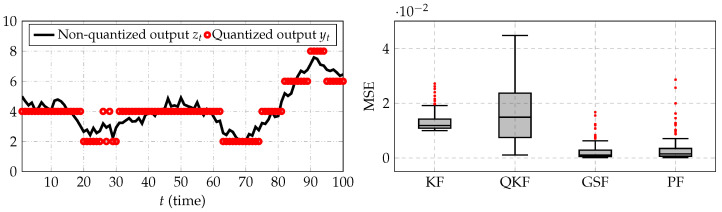
(**Left**) The quantized and nonquantized measurements; (**right**) the MSE between the true and estimated filtered state. KF, QKF, GSF, and PF stand for the Kalman filter, quantized Kalman filter, Gaussian sum filter, and particle filter, respectively.

A linearized model of this system can be found in [78]. Here, it was assumed that $h_{2}$ can be measured and $h_{1}$ cannot. The discrete-time version of the model in [78] with sample time 0.1 s was considered:

(76)xt+1= 0.99590.00410.00410.9959xt+0.0998−0.00020.0002−0.0998ut+wt,(77)zt= 01.0xt+vt,

where $x_{t}={\left[h_{1}\;\;h_{2}\right]}^{T}$ and $u_{t}={\left[f_{1}-k\;\;f_{2}+k\right]}^{T}$ with k=0.4111. The sensor measures $h_{2}$ vary its resistance in discrete steps, with minimum and maximum values $\beta_{1}=2$ and $\beta_{L}=10$. To simulate this system, it was considered that: $w_{t}∼N_{w_{t}}\left(0,0.001I_{2}\right)$, $v_{t}∼N_{v_{t}}\left(0,0.0001\right)$; the input ${\left[f_{1}\;\;f_{2}\right]}^{T}$ was drawn from $N\left({\left[10\;\;2\right]}^{T},10I_{2}\right)$; the initial condition $x_{1}∼N_{x_{1}}\left({\left[10\;\;5\right]}^{T},0.01I_{2}\right)$. For this example, 100 Monte Carlo runs were simulated. In Figure 9 (left), the output $z_{t}$ that corresponds to the values of $h_{2}$ that are nonquantized and the output $y_{t}$ that corresponds to the measurements given by the sensor (for one of the Monte Carlo runs) are shown. In this figure, the level of quantization in the measurements can be observed. In Figure 9 (right), the MSE between the true and estimated state is shown. It was observed that the proposal presented in this paper, the Gaussian sum filter, yielded the most accurate estimation of $h_{1}$, followed by the particle filter, Kalman filter, and quantized Kalman filter. In this example, K=50 and 1000 particles were used to implement the Gaussian sum filter and particle filter, respectively.

In this example, a relatively high number of Gaussian components (K=50) were required to obtain a good estimation of the filtering and smoothing distributions, and hence of the state. This produced an increment in the execution time since the Gaussian sum reduction algorithm needed more time to deal with a high number of Gaussian components in every iteration. This resulted in similar execution times for our proposed algorithm and the traditional particle filter. However, the execution time of the Gaussian sum filter and smoother was smaller than the execution time of the ground truth (particle filter and smoother with 20,000 particles).

## 5. Conclusions

In this paper, Gaussian sum filtering and smoothing algorithms for linear-time-invariant state-space systems with quantized output data were developed. An approximation of the integral equation that defines the probability mass function of the quantized data given the current state, $p(y_{t}|x_{t})$, using Gaussian quadrature was considered. This approximation naturally yielded an explicit mathematical model with a GMM structure for this probability function. Using the approximation of $p(y_{t}|x_{t})$ summarized in Theorem 1, it was possible to solve in closed form the general equations of filtering and smoothing to deal with quantized data. This fact allowed for a closed-form expression of the system state estimators given the quantized data ${\hat{x}}_{t|t}$ and ${\hat{x}}_{t|N}$. Via numerical simulations, it was shown that approximating $p(y_{t}|x_{t})$ with a small number of Gaussian components was adequate, yielding an approximation comparable to the true filtering and smoothing PDFs given by the particle approach (using a large number of particles, namely 20,000 particles). This reduced number of Gaussian components allowed for a low computational load, especially when the system order increased. In addition, our results showed overall less computational load for our proposed techniques since the number of Gaussian components was considerably less than the number of particles used in particle filtering and smoothing.

The proposed Gaussian sum filtering and smoothing algorithms can be utilized, in principle, to develop system identification algorithms and control strategies having quantized output measurements.

## Figures and Tables

**Table 1:** Integral limits and parameters of Theorem 1. ILQ and FLQ mean infinite- and finite-level quantizers, respectively.

	FLQ: $y_{t}=\beta_{1}$	ILQ: $y_{t}=\beta_{i}$ with i∈I in (7) FLQ: $y_{t}=\beta_{i}$ with i=2,…,L−1	FLQ: $y_{t}=\beta_{L}$
$a_{t}$	−∞	$q_{i-1}-Cx_{t}-Du_{t}$	$q_{L-1}-Cx_{t}-Du_{t}$
$b_{t}$	$q_{1}-Cx_{t}-Du_{t}$	$q_{i}-Cx_{t}-Du_{t}$	*∞*
$\varsigma_{t}^{\tau }$	$2\omega_{\tau }/{\left(1+\psi_{\tau }\right)}^{2}$	$\omega_{\tau }\left(q_{i}-q_{i-1}\right)/2$	$2\omega_{\tau }/{\left(1+\psi_{\tau }\right)}^{2}$
$\eta_{t}^{\tau }$	$-\left(1-\psi_{\tau }\right)/\left(1+\psi_{\tau }\right)$	$\psi_{\tau }\left(q_{i}-q_{i-1}\right)/2$	$\left(1-\psi_{\tau }\right)/\left(1+\psi_{\tau }\right)$
$\mu_{t}^{\tau }$	$-q_{1}$	$-(q_{i}+q_{i-1})/2$	$-q_{L-1}$

**Table 2:** Time in seconds required to perform the smoothing algorithm for the scalar system. *Par(K)* represents the number of particles (labeled in Figure 7) that produce a similar result to the Gaussian sum smoother with *K* components. KS, QKS, GSS, and PS stand for the Kalman smoother, quantized Kalman smoother, Gaussian sum smoother, and particle smoother, respectively.

	KS	QKS		GSS				PS		
$\Delta_{q}$	-	-		K=6	K=8	K=10		Par(6)	Par(8)	Par(10)
7	0.0379	0.1494		0.4158	0.4221	0.4735		2.0420	3.3538	4.0462
5	0.0048	0.0044		0.2695	0.3706	0.4588		3.0074	3.5665	4.0192
3	0.0033	0.0043		0.2609	0.3491	0.4618		4.2471	5.5501	6.3408

## Data Availability

No new data were created or analyzed in this study. Data sharing is not applicable to this article.

## Abbreviations

The following abbreviations are used in this manuscript:

PDF/PMF:	Probability density/mass function
GMM:	Gaussian mixture model
GSF/GSS:	Gaussian sum filter/smoother
PF/PS:	Particle filter/smoother
KF/KS:	Kalman filter/smoother
QKF/QKS:	Quantized Kalman filter/smoother
FLQ/ILQ:	Finite-/infinite-level quantizer
MSE:	Mean squared error
$S_{\mathrm{red}}$:	Number of Gaussian components kept after the reduction procedure
GT:	Ground truth obtain by using the particle filter/smoother with a large number
	of particles
K:	Order of the polynomials in the Gauss–Legendre quadrature

## Appendix A. Technical Lemmata

**Lemma** **A1.**
*The PDF $N_{y}\left(Cx+\mu ,R\right)$ of the random variable $Y\in R^{p}$ can be rewritten as follows:*

(A1)
$$N_{y}\left(Cx+\mu ,R\right)=\frac{1}{\sqrt{\det\left\{2\pi R\right\}}}\exp\left\{-\frac{1}{2}\left(x^{T}Fx-2G^{T}x+H\right)\;\right\},$$

*where $F=C^{T}R^{-1}C$, $G^{T}={\left(y-\mu \right)}^{T}R^{-1}C$, and $H={\left(y-\mu \right)}^{T}R^{-1}\left(y-\mu \right)$.*

**Proof.** Directly expand the exponential argument and reorder the terms in the variable *x*. □

**Lemma** **A2.**
*The PDF $N_{x}\left(Aw+\nu ,Q\right)$ of the random variable $X\in R^{n}$ can be rewritten as follows:*

(A2)
$$N_{x}\left(Aw+\nu ,Q\right)=\frac{1}{\sqrt{\det\left\{2\pi Q\right\}}}\exp\left\{-\frac{1}{2}\left(x^{T}Q^{-1}x-2J^{T}x+L\right)\;\right\},$$

*where $J^{T}={\left(Aw+\nu \right)}^{T}Q^{-1}$ and $L={\left(Aw+\nu \right)}^{T}Q^{-1}\left(Aw+\nu \right)$.*

**Proof.** Directly expand the exponential argument and reorder the terms in the variable *x*. □

**Lemma** **A3.** *Consider the backward filter function $p(y_{t:N}|x_{t})$ given in* (42). *Its GMM structure is given by:*

(A3) $$p\left(y_{t:N}|x_{t}\right)=\sum_{k=1}^{S_{t|t}}\sigma_{t|t}^{k}N_{x_{t}}\left(\nu_{t|t}^{k},\Omega_{t|t}^{k}\right),$$

*where $\sigma_{t|t}^{k}={\bar{\sigma }}_{t|t}^{k}/\sum_{s=1}^{S_{t|t}}{\bar{\sigma }}_{t|t}^{s}$ is the normalized mixing weight and $\nu_{t|t}^{k}={\left(F_{t|t}^{k}\right)}^{-1}G_{t|t}^{k}$ and $\Omega_{t|t}^{k}={\left(F_{t|t}^{k}\right)}^{-1},$ are the mean vector and the covariance matrix, respectively, with:*

(A4) $${\bar{\sigma }}_{t|t}^{k}=\frac{\epsilon_{t|t}^{k}\lambda_{t|t}^{k}\exp\left\{-\frac{1}{2}\left(H_{t|t}^{k}-G_{t|t}^{kT}{\left(F_{t|t}^{k}\right)}^{-1}G_{t|t}^{k}\right)\right\}}{{\left(2\pi \right)}^{-\frac{n}{2}}\sqrt{\det\left\{F_{t|t}^{k}\right\}}}.$$

*On the other hand, consider the GMM structure given by* (54). *Then, the backward filter form in* (42) *is obtained using the following:*

(A5) $$\begin{array}{cc} S_{t|t} & =S_{\mathrm{red}},\\ F_{t|t}^{k} & ={\left(U_{t|t}^{k}\right)}^{-1}, \end{array}\;\begin{array}{cc} \epsilon_{t|t}^{k} & =\delta_{t|t}^{k},\\ G_{t|t}^{kT} & ={\left(z_{t|t}^{k}\right)}^{T}{\left(U_{t|t}^{k}\right)}^{-1}, \end{array}\;\begin{array}{cc} \lambda_{t|t}^{k} & ={\left(\det\left\{2\pi U_{t|t}^{k}\right\}\right)}^{-1/2},\\ H_{t|t}^{k} & ={\left(z_{t|t}^{k}\right)}^{T}{\left(U_{t|t}^{k}\right)}^{-1}\left(z_{t|t}^{k}\right). \end{array}$$

**Proof.** Directly by using Lemma A1. □

## Appendix B. Quantities to Perform the Gaussian Sum Filter

For each two-tuple (τ,ℓ), where τ=1,…,K and $\ell =1,\ldots ,M_{t|t-1}$, let *k* be a new index, so that k=(ℓ−1)K+τ. Then:

(A6)γt|tk=γ¯t|tk∑s=1Mt|tγ¯t|ts,(A7)x^t|tk=x^t|t−1ℓ+Ktℓ(ηtτ−κtℓτ),(A8)Σt|tk=(I−KtℓC)Σt|t−1ℓ,

are the weights, means, and covariance matrices of the measurement-update equation in the Gaussian sum filter, with the following definitions:

(A9)γ¯t|tk=ςtτγt|t−1ℓNηtτκtℓτ,Vtℓ,(A10)Ktℓ=Σt|t−1ℓCTVtℓ−1,(A11)κtℓτ=Cx^t|t−1ℓ+Dut+μtτ,(A12)Vtℓ=R+CΣt|t−1ℓCT,

where $\varsigma_{t}^{\tau }$, $\eta_{t}^{\tau }$, and $\mu_{t}^{\tau }$ are defined in Table 1, and:

(A13)γt+1|tk=γt|tk,(A14)x^t+1|tk=Ax^t|tk+But,(A15)Σt+1|tk=Q+AΣt|tkAT,

are the weights, means, and covariance matrices of the time-update equation in the Gaussian sum filter.

## Appendix C. Quantities to Perform the Backward Filter

For each two-tuple (τ,ℓ), where τ=1,…,K and $\ell =1,\ldots ,S_{t|t+1}$, let *k* be a new index, so that k=(ℓ−1)K+τ. Then:

(A16)ϵt|tk=ςtτϵt|t+1ℓ,(A17)λt|tk=det2πR−1/2λt|t+1ℓ,(A18)θtτ=ηtτ−Dut−μtτ,(A19)Ft|tk=Ft|t+1ℓ+CTR−1C,(A20)Gt|tkT=Gt|t+1ℓT+θtτTR−1C,(A21)Ht|tk=Ht|t+1ℓ+θtτTR−1θtτ.

are the quantities needed to compute the backward-measurement-update equation in the backward filter, where $\varsigma_{t}^{\tau }$, $\eta_{t}^{\tau }$, and $\mu_{t}^{\tau }$ are defined in Table 1, and:

(A22)ϵt|t+1k=ϵt+1|t+1k,(A23)λt|t+1k=detQdetFqk−1/2λt+1|t+1k,(A24)Ft|t+1k=ATMqkA,(A25)Gt|t+1kT=Gt+1|t+1kTFqk−1Q−1A−utTBTMqkA,(A26)Ht|t+1k=Ht+1|t+1k−Gt+1|t+1kTFqk−1Gt+1|t+1k+utTBTMqkBut−2utTBTQ−1Fqk−1Gt+1|t+1k,

are the quantities needed to compute the backward prediction equation in the backward filter, where $F_{qk}=F_{t+1|t+1}^{k}+Q^{-1}$ and $M_{qk}=Q^{-1}-Q^{-1}F_{qk}^{-1}Q^{-1}$.

## Appendix D. Quantities to Perform the Gaussian Sum Smoother

For each two-tuple (τ,ℓ), where $\tau =1,\ldots ,M_{t|t-1}$ and $\ell =1,\ldots ,S_{\mathrm{red}}$, let *k* be a new index, so that $k=\left(\ell -1\right)M_{t|t-1}+\tau$. Then:

(A27)ϵt|Nk=ϵ¯t|Nk∑s=1St|Nϵ¯t|Ns,(A28)x^t|Nk=(Ltℓτ)−1ρtℓτ,(A29)Σt|Nk=(Ltℓτ)−1,

are the weights, means, and covariance matrices of the smoothing PDF $p(x_{t}|y_{1:N})$, where:

(A30)ϵ¯t|Nk=γt|t−1τδt|tℓexp−12ϕ1tℓ+ϕ2tτ−ϕ3tℓτ(2π)n2detLtℓτdetUt|tℓdetΣt|t−1τ,(A31)ρtℓτ=Ut|tℓ−1zt|tℓ+Σt|t−1τ−1x^t|t−1τ,(A32)Ltℓτ=Ut|tℓ−1+Σt|t−1τ−1,(A33)ϕ3tℓτ=ρtℓτTLtℓτ−1ρtℓτ,(A34)ϕ1tℓ=zt|tℓTUt|tℓ−1zt|tℓ,(A35)ϕ2tτ=x^t|t−1τTΣt|t−1τ−1x^t|t−1τ,

$\gamma_{t|t-1}^{\tau }$, ${\hat{x}}_{t|t-1}^{\tau }$ and $\Sigma_{t|t-1}^{\tau }$ are obtained from the time-update step of Theorem 2 (34), and $\delta_{t|t}^{\ell }$, $z_{t|t}^{\ell }$, and $U_{t|t}^{\ell }$ are obtained from the reduced measurement-update step of the backward-filtering algorithm in (54).

## Appendix E. Quantities to Compute *p*(*x*_*t*+1_, *x_t_*|*y*_1:*N*_)

For each two-tuple (τ,ℓ), where $\tau =1,\ldots ,M_{t|t}$ and $\ell =1,\ldots ,S_{t+1|t+1}$, let *k* be a new index, so that $k=\left(\ell -1\right)M_{t|t}+\tau$. Then:

(A36)αk=α¯k∑s=1St,t+1α¯s,(A37)χ^t|Nk=(Ftℓτ)−1Gtℓτ,(A38)Et|Nk=(Ftℓτ)−1,

are the weights, means, and covariance matrices of the joint PDF $p(x_{t+1},x_{t}|y_{1:N})$, where:

(A39)α¯k=γt|tτϵt+1|t+1ℓλt+1|t+1ℓStℓτdetQdetFtℓτdetΣt|tτ,(A40)Stℓτ=exp−12Htℓτ−GtℓτTFtℓτ−1Gtℓτ,(A41)Ftℓτ=Q−1+Ft+1|t+1ℓ−Q−1A−ATQ−1Σt|tτ−1+ATQ−1A,(A42)GtℓτT=Gt+1|t+1ℓT+utTBTQ−1JtτT−utTBTQ−1A,(A43)Htℓτ=Ht+1|t+1ℓ+Ltτ+utTBTQ−1But,

with:

(A44)JtτT=x^t|tτTΣt|tτ−1,(A45)Ltτ=x^t|tτTΣt|tτ−1x^t|tτ,

$\gamma_{t|t}^{\tau }$, ${\hat{x}}_{t|t}^{\tau }$, and $\Sigma_{t|t}^{\tau }$ are obtained from the measurement-update step in Theorem 2, and $\epsilon_{t+1|t+1}^{\ell }$, $\lambda_{t+1|t+1}^{\ell }$, $F_{t+1|t+1}^{\ell }$, $G_{t+1|t+1}^{\ell T}$, and $H_{t+1|t+1}^{\ell }$ are obtained from the reduced backward-measurement-update step in Theorem 3.
